# Tripartite motif containing 24 regulates cell proliferation in colorectal cancer through YAP signaling

**DOI:** 10.1002/cam4.3310

**Published:** 2020-07-17

**Authors:** Wenlin Xie, Yingqiang Zhang, Bingyang Wang, Yuting Hu, Bohui Zhan, Fangqiang Wei, Jianming Tang, Jiayan Lian

**Affiliations:** ^1^ Department of Pathology The Seventh Affiliated Hospital of Sun Yat‐Sen University Shenzhen Guangdong P.R. China; ^2^ Department of Interventional Radiology The Seventh Affiliated Hospital of Sun Yat‐Sen University Shenzhen Guangdong P.R. China; ^3^ Department of Hepatobiliary and Pancreatic Surgery Zhejiang Provincial People’ s Hospital People’s Hospital of Hangzhou Medical College Hangzhou Zhejiang P.R. China; ^4^ Department of Radiation Oncology Zhejiang Provincial People’ s Hospital People’ s Hospital of Hangzhou Medical College Hangzhou Zhejiang P.R. China

**Keywords:** Cell proliferation, Colorectal cancer, TRIM24, YAP

## Abstract

The protein, tripartite motif containing 24 (TRIM24) is a member of the TRIM protein family, and acts as a critical co‐regulator of multiple nuclear receptors. TRIM24 is dysregulated in many cancers, including colorectal carcinoma. However, its biological functions and molecular mechanisms with respect to colorectal carcinoma are still largely unknown. In the current study, we found that TRIM24 promotes YAP signaling for driving cell proliferation in colorectal cancer. TRIM24 was significantly upregulated in colorectal carcinoma, and its expression was negatively correlated with the survival of patients. Depletion of TRIM24 impaired the ability of the cancer cells to proliferate and form colonies. Furthermore, this study also revealed the mechanism underlying the recruitment of TRIM24 by the DANCR/KAT6A complex, which is bound to acetylated lysine 23 of histone H3 (H3K23), resulting in binding to the YAP promoter and activation of YAP transcription that ultimately enhances the proliferation of colorectal cancer cells. Our results revealed a novel mechanism involving TRIM24‐YAP signaling for the regulation of colorectal cancer. We also identified TRIM24 as a potential therapeutic molecule for targeting colorectal cancer.

## Graphical abstract

Here, we evaluated TRIM24 expression in colorectal cancer, and revealed a positive correlation between TRIM24 expression level and survival prognosis. TRIM24 promotes cell proliferation via YAP signaling. Finally, potential mechanism of DANCR/KAT6A complex on TRIM24 response was explored.

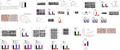

## 1 INTRODUCTION

Colorectal carcinoma is the third most common malignancy worldwide, and accounts for a significant proportion of cancer‐related deaths.^1^ Despite significant advances being made in the development of treatments involving molecular targets, the median survival of patients with colorectal cancer still remains low.^2^ This outcome has fostered major efforts aimed at discovering novel molecular targets for the treatment of colorectal cancer.

TRIM24 is an important member of the transcription intermediary factor (TIF) family.^3^ It consists of a C‐terminal tandem PHD finger domain, a conserved N‐terminal tripartite motif domain, and a bromodomain.^4^ TRIM24 has been shown to be oncogenic, and its expression is positively associated with the poor survival of patients with various cancers, such as bladder cancer,^5^ gastric cancer,^6^ human HCC,^7^ nonsmall cell lung cancer,^8^ breast cancer,^9^ head and neck cancer.^10^ Previous research found that upregulation of TRIM24 was significantly associated with poor survival of colorectal cancer patients.^11^ Another study reported that knockdown of TRIM24 significantly inhibited the growth of colorectal cancer cells, and induced apoptosis.^12^ TRIM24 has also been identified as targeting p53 in human breast cancer.^13^ In glioblastoma, it has been shown to upregulate PI3K/AKT signaling by interacting with KAT6A.^14^ The TRIM24 gene has been shown to be targeted by NCK1‐AS1 in glioma cells.^15^ However, the function of TRIM24 in colorectal cancer remains unclear.

Several studies have demonstrated that abnormal expression of the Hippo/YAP signaling pathway plays a critical role in tumorigenesis, including colorectal cancer.^16^, ^17^ Recent evidence has shown that YAP forms complexes with certain transcription factors, and then drives tumor initiation and progression.^18^ YAP phosphorylation, a critical event in YAP signaling, is a key downstream transducer of the Hippo pathway.^19^ Lysine methylation is also involved in YAP activation.^20^ However, the precise mechanisms by which YAP activation mediates the development of colorectal cancer are still largely unknown.

We evaluated the expression of TRIM24 in colorectal cancer patients, and found a positive correlation between the expression pattern of the protein and the clinical outcomes. We showed that TRIM24 promotes cell proliferation through YAP signaling. Finally, we explored the potential role of the DANCR/KAT6A complex in mediating the TRIM24 response.

## 2 MATERIALS AND METHODS

### 2.1 Clinical samples

A total of 80 clinical colorectal tumor tissues and paired peri‐tumoral tissues were obtained from the Seventh Affiliated Hospital of Sun Yat‐Sen University between May 2018 and January 2020. None of the patients had received chemotherapy or radiotherapy before biopsy. This study was approved by the Institutional Ethical Review Boards of the Seventh Affiliated Hospital of Sun Yat‐Sen University.

### 2.2 Cell lines

HCT116, LOVO, SW620, HT29, and NM460 cell lines were purchased from the Cell Bank of the Chinese Scientific Academy (Shanghai, China). NCM460 cells were cultured in DMEM/F12 (Invitrogen, Carlsbad, CA) supplemented with 20 ng/mL epidermal growth factor (EGF), 5% horse serum, 100 ng/mL cholera toxin, 0.5 μg/mL hydrocortisone, 100 μg/mL penicillin‐streptomycin, and 10 μg/mL insulin. All colorectal cancer cells were cultured in Dulbecco's modified Eagle's medium (Invitrogen, Carlsbad, CA) containing 10% fetal bovine serum. All cells were cultured in an incubator with a 5% CO2 atmosphere at 37°C.

### 2.3 Chromatin immunoprecipitation (ChIP)

ChIP was conducted according to the manufacturer's instructions. Approximately 1 × 10^7^ cells were fixed using 1% formaldehyde in a 100 mm culture dish for cross‐linking proteins to DNA. The cell lysates were sonicated to shear DNA to sizes ranging from 500 to 1,000 bp. The chromatin supernatants were divided into equal aliquots, and 1ug of anti–TRIM24 or H3K23ac was added. Anti‐IgG was used as a control. The samples were rocked overnight at 4°C. PCR was preformed upon decross‐linking the protein and DNA complexes to give rise to free DNA. The primers used for PCR are shown in Table S1.

### 2.4 Luciferase assay

Following the manufacturer's protocol, the pGL3.0‐Basic luciferase reporter vectors containing the wild‐type and TRIM24 binding sites mutant‐type YAP promoter were co‐transfected with or without TRIM24 using Lipofectamine™ 2000 Transfection Reagent (Thermo Fisher). We used pRL‐TK Renilla plasmid (Promega) as a negative control. After 48 hours, a dual‐luciferase assay (Promega) was performed in accordance with the manufacturer's recommendations.

### 2.5 Western blotting (WB) and immunoprecipitation (IP)

WB and IP were conducted as previously described.^26^ In brief, the cells were lysed in the required amount of IP lysis buffer (150 mmol/L NaCl, 20 mmol/L.

Tris‐HCl, 5 mmol/L NaF, pH 7.5, 2 mmol/L Na3VO4, 1% Triton X‐100, 1x protease inhibitor cocktail), and 1 mmol/L EDTA at 4°C for 30 minutes. The lysates were then cleared by centrifugation. Equal amounts of cell lysates were subsequently immunoprecipitated with the target antibodies and protein G‐agarose control beads (Invitrogen). Finally, western blotting was performed using a standard protocol.

### 2.6 Cell proliferation and colony formation assay

The cell proliferation and colony formation assay were conducted as described previously ^26^. In brief, 1,000 cells were seeded into 96‐well plates and incubated for three days at 37°C. Cell proliferation assays were performed using WST‐1 assay kits (Roche). For the colony formation assay, 400 cells were seeded into each well of six‐well plates. After incubation for one to two weeks, the colonies were fixed with 4% paraformaldehyde and stained with 1% crystal violet. Finally, the stained colony numbers were scored and analyzed.

### 2.7 Western blotting

Western blots were performed as described previously.^27^ Antibodies used for western blotting were as follows: β‐actin (#4970, Cell Signaling Technology, 1:1,000), KAT6A (ABP59003, Abbkine, 1:1,000,), YAP (#14074, Cell Signaling Technology, 1:1,000,), and H3K23ac (#8173S Cell Signaling Technology, 1:1,000,), AXL (ab226927, Abcam, 1:1,000), and CTGF (ab6992, Abcam, 1:1,000).

### 2.8 Plasmid preparation

NCM460 cells were used to amplify the DNA sequences of TRIM24 and YAP using RT‐PCR. Those DNA sequences were then subcloned into pcDNA3.3 or pLVX‐Puro vectors (Clontech), respectively. The YAP promoter was subcloned into the pGL3‐basic luciferase reporter vector.

### 2.9 shRNA‐knockdown and transfection assays

TRIM24, DANCR, and KAT6A shRNA sequences were purchased from GeneChem (Shanghai, China). The DNA sequences and the packaging plasmids were transfected into HEK293 cells. After transfection for 48 and 72 hours, the cell supernatant was filtered through a 0.22‐µm membrane (Millipore). The SW620 and HT29 cells were infected using lentiviruses expressing shRNAs or shGFP control (8 µg/mL polybrene). The infected cells were selected using 5 μg/ml puromycin. Multiple monoclonal cultures were screened for shRNAs using and RT‐PCR and Western blotting.

### 2.10 IHC of human colorectal cancer specimens

The paraffin‐embedded human colorectal cancer specimens were sectioned and subjected to immunohistochemistry using antibodies against TRIM24 (1:100). Based on the intensity of the staining (0 = no staining; 1 = weak staining; 2 = moderate staining; 3 = strong staining) and the proportion of the stained cells (0 = 0%; 1 = 1%‐25%; 2 = 25%‐50%; 3 = 50%‐75%; and 4 = 75%‐100%); each sample was assigned a score. Negative control slides without primary antibodies were included. Low TRIM24‐expressing tumors had staining scores of 0 to 3, while high TRIM24‐expressing tumors had staining scores of 4 to 7. Scoring was conducted by three individuals who were blinded to the clinical characteristics.

### 2.11 Tumorigenesis studies

Female BALB/c nude mice approximately 4‐week‐old (SLAC, Shanghai, China) were divided into two groups and housed under pathogen‐free conditions. SW620 cells were collected and washed twice with serum‐free medium. After resuspension in serum‐free DMEM and mixing 1:1 with Matrigel (Becton‐Dickinson), SW620 cells (3 × 10^6^) were implanted subcutaneously into the right flank of each nude mouse. Mice were euthanized and tumor weights were recorded. These animal experimental procedures were approved by the Institutional Animal Care and Use Committee of the Seventh Affiliated Hospital of Sun Yat‐Sen University.

### 2.12 Statistical analysis

We conducted statistical analyses using GraphPad Prism version 5.0. One‐way analysis of variance (ANOVA) with Newman‐Keuls post‐tests, or unpaired two‐tailed Student's *t‐*tests were used to determine the significant differences between experimental groups Correlation was calculated using Spearman's rank correlation coefficients. Log‐rank tests and the Kaplan‐Meier method were used for survival analysis. A two‐sided P value of < 0.05 was considered to indicate significance, unless otherwise stated.

## 3 RESULTS

### 3.1 TRIM24 is upregulated in colorectal carcinoma and is negatively correlated with patient survival

**Figure 1:**
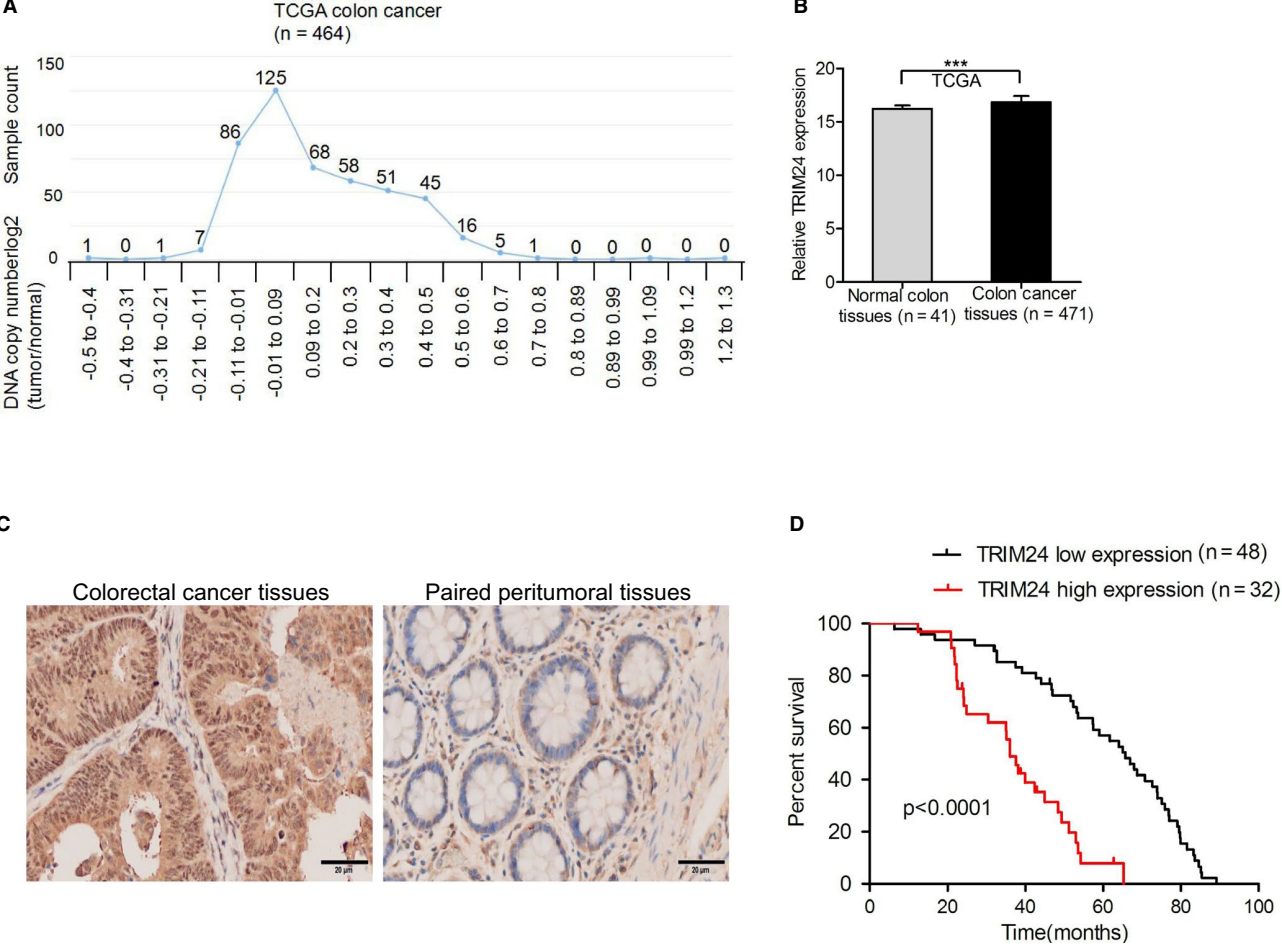
TRIM24 is upregulated and negatively correlated with survival prognosis in colorectal cancer. (A), Copy number analysis of TRIM24 in the TCGA colon cancer dataset. The data were downloaded from http://xena.ucsc.edu/public‐hubs. (B), TRIM24 mRNA expression in tumor and normal specimens. TRIM24 mRNA expression was obtained from the TCGA dataset from http://xena.ucsc.edu/public‐hubs. (C), A total of 80 clinical colorectal cancer and paired peri‐tumoral tissues were analyzed by immunohistochemistry (IHC). Representative images of TRIM24 expression in serial sections from colorectal cancer tissues are shown. Scale bars: 50 μm. (D), Kaplan‐Meier analyses of TRIM24 high or low expression patients categorized based on IHC. Error bars show ± SD. ***P < 0.001. The data represent the results from three independent experiments

DNA copy numbers of TRIM24 were analyzed using data from the TCGA colorectal cancer dataset (http://xena.uscs.edu/public‐hubs). The gene was amplified in 52.6% (244/464) of the tumors (log2 > 0.09, tumor vs normal) relative to that in normal colon tissues (Figure 1A). These results indicated that TRIM24 is overexpressed in colorectal carcinoma. Higher expression levels of TRIM24 mRNA were also observed in the TCGA colorectal cancer dataset (http://xena.uscs.edu/public‐hubs) (Figure 1B). To validate this finding, we evaluated the expression of TRIM24 in tumor and paired peri‐tumoral tissues at the protein level. As shown in Figure 1C, we found that TRIM24 protein was more highly expressed in colorectal tumor tissues than in paired peri‐tumoral tissues. Kaplan‐Meier survival analysis indicated that patients who had low levels of TRIM24 had better prognoses than those with high TRIM24 levels (Figure 1D). These results showed that TRIM24 is significantly upregulated in colorectal carcinoma, and is negatively correlated with survival prognosis.

### 3.2 TRIM24 promotes cell proliferation in colorectal cancer

**Figure 2:**
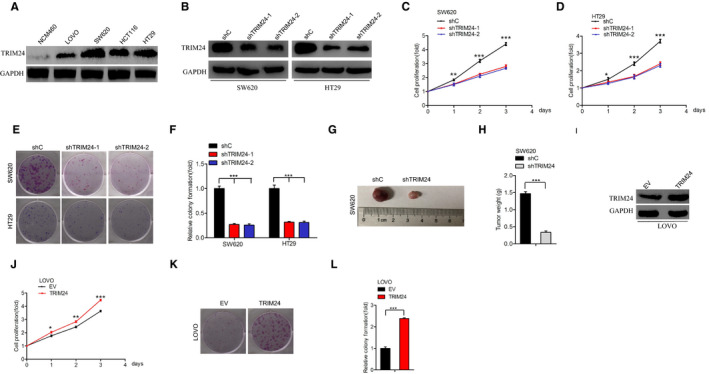
TRIM24 promotes cell proliferation in colorectal cancer. (A), Expression of the TRIM24 protein in the colorectal cancer and colonic epithelial cell lines. (B), Effects of DANCR depletion in colorectal cancer cells. (C‐D), Effect of DANCR depletion on the proliferation of colorectal cancer cells. (E), Depletion of DANCR inhibits the formation of cell colonies. (F), Quantification of the data shown in (E). (G), Representative images depicting shTRIM24‐mediated inhibition of subcutaneous tumor generation by SW620 cells. Tumors were harvested three to four weeks after cell implantation. Data are from three independent experiments, with five mice per group. (H), Quantification of tumor weight in (G). (I), Effect of DANCR overexpression in colorectal cancer cells. (J), Effects of DANCR overexpression on the proliferation of colorectal cancer cells. (K), DANCR overexpression enhances the formation of cell colonies. (L), Quantification of the data shown in (K). Error bars show ± SD. *P < .05. **P < .01. ***P < .001. The data represent the results from three independent experiments

To investigate the molecular function of TRIM24 in colorectal cancer, we first evaluated TRIM24 expression in a colonic epithelial cell line, NCM460, and four colorectal cancer cell lines: LOVO, SW620, HT29, and HCT116. The expression levels of TRIM24 were higher in all colorectal cancer cells than in NCM460 cells (Figure 2A). Next, we depleted TRIM24 in SW620 and HT29 cells (Figure 2B). TRIM24 knockdown significantly suppressed cell proliferation (Figure 2C and D) and colony formation (Figure 2E and F) in vitro. TRIM24 knockdown also suppressed tumorigenicity in vivo (Figure 2G and H). We also overexpressed TRIM24 in LOVO cells (Figure 2I). TRIM24 overexpression promoted cell proliferation (Figure 2J) and colony formation (Figure 2K and L). These results supported the hypothesis that TRIM24 promotes cell proliferation in colorectal cancer.

### 3.3 TRIM24 promotes cell proliferation through YAP

**Figure 3:**
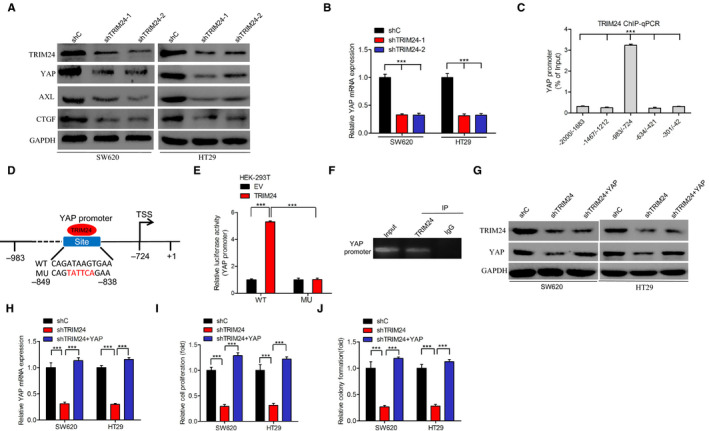
TRIM24 promotes cell proliferation through YAP. A‐B), Effects of TRIM24 knockdown on the expression of YAP at protein (A) and mRNA (B) levels in SW620 and HT29 cells. (C), ChIP‐qPCR showing the binding of TRIM24 to the YAP promoter. (D), Schematic diagram of the putative TRM24‐binding sites in the YAP promoter. (E), Effects of TRIM24 on YAP promoter activity as shown by luciferase assay. (F), TRIM24 binds to the YAP promoter in vivo. (G‐H), YAP overexpression rescues TRIM24 knockdown‐induced inhibition of YAP protein (G) and mRNA (H) expression. (I‐J), YAP overexpression rescues TRIM24 knockdown‐induced inhibition of cell proliferation (I) and colony formation (J). Error bars show ± SD. ***P < .001. The data represent the results from three independent experiments

A previous study showed that YAP strongly promotes the proliferation of colorectal cancer cells.^21^ We depleted the expression of TRIM24 in SW620 and HT29 cells, and found that knockdown of TRIM24 inhibited the expression of YAP at both mRNA and protein level (Figure 3A and B). TRIM24 knockdown also suppressed genes downstream of YAP, such as AXL and CTGF^21^ (Figure 3A). We hypothesize that TRIM24 promotes cancer cell proliferation by activating the transcription of YAP. As shown in Figure 3C, TRIM24 directly binds to the YAP promoter at the −983 to −734 site. Overexpression of TRIM24 markedly increased YAP promoter activity in comparison with the empty vector, while YAP promoter mutation markedly decreased the activity of its promoter, which was activated by TRIM24 overexpression (Figure 3D and E). We performed ChIP‐PCR assays in tumor tissues (Figure 2G **)** to detect interactions between TRIM24 and the YAP promoter in vivo (Figure 3F).

To determine the effect of YAP on TRIM24‐mediated cell growth in colorectal cancer, we overexpressed YAP in TRIM24‐knockdown SW620 and HT29 cells (Figure 3G and H). Although overexpression of YAP rescued TRIM24 depletion‐mediated inhibition of cell growth (Figure 3I) and colony formation (Figure 3J), it did not affect the impairment of TRIM24 protein expression due to knockdown (Figure 3G). These data further support the hypothesis that TRIM24 promotes cell proliferation by promoting YAP transcription.

### 3.4 The DANCR/KAT6A complex upregulates TRIM24 in colorectal carcinoma

**Figure 4:**
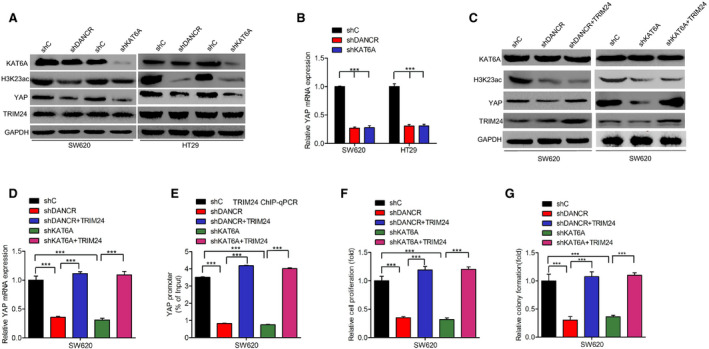
DANCR/KAT6A complex upregulates TRIM24 in colorectal cancer. A‐B), Effects of DANCR or KAT6A knockdown on YAP expression at protein (A) and mRNA (B) levels in SW620 and HT29 cells. (C–D), TRIM24 overexpression rescues the DANCR or KAT6A knockdown‐mediated inhibition of YAP expression at protein (C) and mRNA (D) levels. (E), TRIM24 rescues DANCR or KAT6A knockdown‐mediated inhibition of the binding between TRIM24 and the YAP promoter, as shown by ChIP‐qPCR. (F‐G), TRIM24 rescues DANCR or KAT6A knockdown‐mediated inhibition of (F) and colony formation (G). Error bars show ± SD. ***P < 0.001. The data represent the results from three independent experiments

DANCR and KAT6A, long non‐coding RNA (lncRNA) molecules, have been reported to form a complex which mediates cell proliferation in colorectal cancer.^22^ To gain insights into the precise role of the DANCR/KAT6A complex in mediating the function of TRIM24 in colorectal carcinoma, we inhibited the expression of DANCR or KAT6A in both SW620 and HT29 cells, and found that both mRNA and protein expression of YAP were inhibited (Figure 4A and B). However, TRIM24 expression levels were not affected upon DANCR or KAT6A knockdown (Figure 4A and B). TRIM24 overexpression attenuated the DANCR or KAT6A shRNA‐mediated inhibition of YAP protein and mRNA expression (Figure 4C and D). Overexpression of TRIM24 largely restored the association of TRIM24 with the YAP promoter, an association that was inhibited by DANCR or KAT6A depletion (Figure 4E). TRIM24 overexpression also rescued the DANCR or KAT6A knockdown‐mediated inhibition of cell proliferation and colony formation (Figure 4F and G). Thus, the DANCR/KAT6A complex upregulates TRIM24 in colorectal cancer.

### 3.5 Association of H3K23ac with TRIM24 is critical for YAP expression

**Figure 5:**
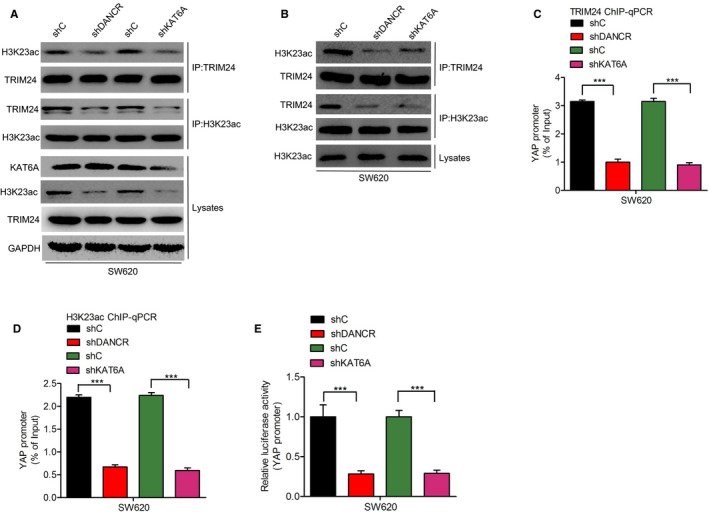
Association of TRIM24 with H3K23ac is critical for YAP expression. (A‐B), DANCR or KAT6A depletion inhibits the association of TRIM24 with H3K23ac. (C‐D), DANCR or KAT6A depletion inhibits the binding of TRIM24 (C) or H3K23ac (D) to the YAP promoter as shown by ChIP‐qPCR. (E), Effect of DANCR or KAT6A depletion on the YAP promoter activity. Error bars show ± SD. ***P < 0.001. The data represent the results from three independent experiments

It is known that KAT6A promotes the binding of H3K23ac to TRIM24 in glioma cells^23^. We therefore investigated the effect of DANCR or KAT6A depletion on the interactions between H3K23ac with TRIM24 in SW620 colorectal cells. Figure 5A and B show that depletion of DANCR or KAT6A significantly decreased the association of TRIM24 with H3K23ac, suggesting that the DANCR/KAT6A complex is critical for this interaction. We also found, using ChIP‐qPCR assays, that DANCR or KAT6A knockdown inhibited the direct combination of TRIM24 and H3K23ac with the YAP promoter (Figure 5C and D). Luciferase assays also revealed that depletion of DANCR or KAT6A inhibits H3K23ac with TRIM24‐mediated transcription of YAP promoter (Figure 5E). These results indicate that the binding of H3K23ac with TRIM24 is critical for YAP expression in colorectal cancer.

### 3.6 TRIM24 is positively correlated with YAP in clinical colorectal cancer specimens

**Figure 6:**
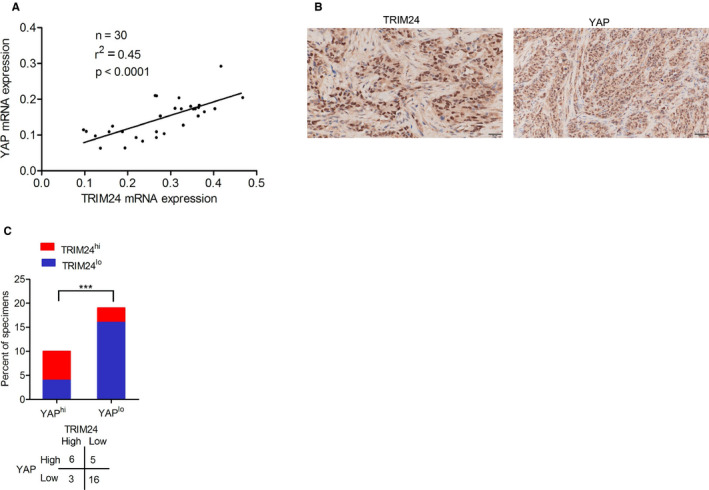
TRIM24 positively correlates with YAP expression in clinical colorectal cancer specimens. (A), Correlation analysis of YAP and TRIM24 mRNA expression. (B), Representative images of immunohistochemical staining of YAP and TRIM24 proteins. Scale bars: 50 μm. (C), Correlation analysis between expression of YAP and TRIM24 proteins as shown in (B). Error bars show ± SD. ***P < .0001. The data represent the results from three independent experiments

To evaluate the clinical implications of our results, we measured the expression levels of TRIM24 and YAP in clinical colorectal cancer specimens. We collected 30 paraffin‐embedded colorectal cancer specimens. As shown in Figure 6A, Spearman's rank correlation analysis found that the mRNA levels of TRIM24 were significantly correlated with those of YAP. Immunohistochemical (IHC) staining also revealed that TRIM24 protein levels were positively correlated with those of YAP (Figure 6B and C **)**. Therefore, we concluded that TRIM24 is positively correlated with YAP in colorectal cancer.

## 4 DISCUSSION

In this study, we found that TRIM24 plays a critical role in the proliferation of colorectal cancer cells via YAP signaling. TRIM24 was amplified and upregulated in colorectal carcinoma, and TRIM24 levels were negatively associated with patient survival. TRIM24 is initially activated by the DANCR/KAT6A complex, thereby binding with H3K23ac and subsequently inducing TRIM24‐mediated recruitment of YAP to the chromatin, which ultimately promotes the proliferation of colorectal cancer cells.

Our results indicated that TRIM24 is critical for the development of colorectal cancer. Accumulating evidence has shown that TRIM24 plays an important role in the development of colorectal cancer.^11^, ^12^ Patients who had higher levels of TRIM24 expression showed poorer overall survival than those who had lower levels of TRIM24 expression.^11^ We found that TRIM24 was upregulated in colorectal carcinoma, and demonstrated that its expression is inversely correlated with the survival of colorectal cancer patients. TRIM24 knockdown suppressed cell proliferation and colony formation. Hence, we conclude that TRIM24 is essential to the development of colorectal carcinoma.

We further found that the DANCR/KAT6A complex promoted the association of TRIM24 with H3K23ac, thereby inducing TRIM24‐mediated recruitment of YAP to the chromatin, and promoting proliferation of colorectal cancer cells. A previous study revealed that DANCR interacts with KAT6A, which promotes proliferation of colorectal cancer cells.^23^ TRIM24 was shown to be oncogenic in colorectal cancer,^11^, ^12^ but the relationship between the DANCR/KAT6A complex and TRIM24 in colorectal cancer had not previously been investigated. We found that the DANCR/KAT6A complex upregulated TRIM24, and promoted cell proliferation in colorectal cancer. This result is consistent with that of a previous study, which found that the association of TRIM24 with H3K23ac regulates downstream‐targeted genes in breast cancer,^24^ prostate cancer,^25^ and glioblastoma.^26^ Our data also suggest that DANCR/KAT6A promotes the association between TRIM24 and H3K23ac, thereby enhancing the TRIM24‐mediated recruitment of YAP to the chromatin, resulting in the proliferation of colorectal cancer cells. We found that TRIM24 levels are positively correlated with those of YAP.

In summary, our results indicated that TRIM24 serves as an oncogene in colorectal.

carcinoma. We elucidated a previously unknown molecular mechanism involving DANCR/KAT6A‐mediated association of TRIM24 with H3K23ac, which subsequently results in enhancement of the oncogenic processes associated with the YAP signaling pathway in colorectal cancer. This novel finding potentially provides a strong basis for the development of new treatment options for patients sufferings from colorectal cancer.

## CONFLICT OF INTEREST

All authors declare no conflict of interest.

## AUTHORS' CONTRIBUTIONS

Conception and design: Fangqiang Wei, Jianming Tang, and Jiayan Lian. Development of methodology: Wenlin Xie, and Yingqiang Zhang. Analysis and interpretation of data (e.g., statistical analysis, biostatistics and computational analysis): Fangqiang Wei, Jianming Tang, and Jiayan Lian. Writing, review and/or critical revision of the manuscript: Bingyang Wang, Yuting Hu, and Bohui Zhan. Administrative, technical or material support (i.e., reporting or organizing data, and constructing databases): Wenlin Xie, and Yingqiang Zhang. Study supervision: Jianming Tang, and Jiayan Lian. All authors read and approved the final manuscript.

## Supporting information

Table S1

## Data Availability

The datasets used and/or analyzed during the current study are available from the corresponding author on reasonable request.
