# Supplementary material for: Tripartite motif containing 24 regulates cell proliferation in colorectal cancer through YAP signaling
Source: Cancer Med. 2020 Jul 17;9(17):6367–76. doi: 10.1002/cam4.3310 (PMC7476840; doi:10.1002/cam4.3310)
Supplement: Supplementary file 1 — Table S1 [file CAM4-9-6367-s001.doc]

**Supplementary Table 1** Primers for qRT-PCR assays and ChIP-qPCR assays

| Primer pairs | Sequence |
| --- | --- |
| qRT-PCR,GAPDH | 5’-GGAGCGAGATCCCTCCAAAAT-3’ and  5’-GGCTGTTGTCATACTTCTCATGG-3’ |
| qRT-PCR,YAP | 5’-TAGCCCTGCGTAGCCAGTTA-3’ and  5’- TCATGCTTAGTCCACTGTCTGT-3’ |
| ChIP-qPCR,  YAP promoter  -2000 to -1683 | 5’-CCCACTTTGGTTCCTAA-3’ and  5’-GGGTAATAACACCTACC-3’ |
| ChIP-qPCR,  YAP promoter  -1467 to -1212 | 5’-TTCAGAGTAGTTGATTG-3’and  5’-AATGCACAAGCCCATTCT-3’ |
| ChIP-qPCR,  YAP promoter  -983 to -724 | 5’-CCTCCTTGCCCATTCAT-3’and  5’-AGTGTAACTTATTAAAAGA-3’ |
| ChIP-qPCR,  YAP promoter  -634 to -421 | 5’-ATTACAAAATAAAGTTAAT-3’and  5’-TTCGCCTGGACCTGCGAAA-3’ |
| ChIP-qPCR,  YAP promoter  -301 to -42 | 5’-CCATCGTTTGCGGTTCGCG-3’and  5’-ACTAAAGTTAGGAGGAAGC-3’ |
